# Supplementary material for: Unmixing biological fluorescence image data with sparse and low-rank Poisson regression
Source: Bioinformatics. 2023 Mar 25;39(4):btad159. doi: 10.1093/bioinformatics/btad159 (PMC10081874; doi:10.1093/bioinformatics/btad159)
Supplement: btad159_Supplementary_Data [file btad159_supplementary_data.pdf]

Unmixing Biological Fluorescence Image Data with Sparse and Low-Rank Poisson Regression: Supplementary Material

Ruogu Wang, Alex A. Lemus, Colin M. Henneberry, Yiming Ying, Yunlong Feng, and Alex M. Valm

Contents

1 The Proposed Algorithms for Solving PNMF and SL-PRU i

2 Supplementary Table 1: Salient Characteristics of FISH Probes vi

3 Supplementary Figure 1: Emission Spectra of Endmembers and the Extracted Endmembers vii

4 Supplementary Table 2: Descriptions of Fluorophores viii

5 Supplementary Figure 2: Quantitative Line Scan Analysis on Dental Plaque Smear Images ix

6 Supplementary Movie: SL-RPU Unmixed Dental Plaque Smear x

References xi

1 The Proposed Algorithms for Solving PNMF and SL-PRU

We first present the developed algorithms for solving the endmember extraction problem through PNMF and for solving the abundance estimation problem through SL-PRU, respectively. To this end, we first recall that the endmember $\mathbf{m} \in \mathbb{R}_+^C$ extracted from a reference image $\mathbf{Y_m} \in \mathbb{R}_+^{C\times N}$ and the corresponding abundances $\mathbf{a} \in \mathbb{R}_+^N$ are obtained through Poisson Nonnegative Matrix Factorization (PNMF):

$$\min_{\mathbf{m}\in\mathbb{R}_+^C, \mathbf{a}\in\mathbb{R}_+^N} \mathbf{1}_C^\top \left[\mathbf{m}\mathbf{a}^\top - \mathbf{Y_m} \circ \log\left(\mathbf{m}\mathbf{a}^\top\right)\right] \mathbf{1}_N, \tag{1}$$

where $\mathbf{1}_C$ and $\mathbf{1}_N$ are vectors of length C and N whose entries are all 1 and $\circ$ denotes element-wise multiplication. PNMF (1) can be solved by *multiplicative update algorithm* (Lee and Seung, 2000) which is a diagonally rescaled version of gradient descent. Denoting any variable $\mathbf{X}$ at the t-th, iteration as $\mathbf{X}^{(t)}$ and the maximum norm as $\|\cdot\|_\infty$, the pseudocode of the multiplicative update algorithm is provided in Algorithm 1. The abundance vector $\mathbf{a}$ and the endmember vector $\mathbf{m}$ are initialized with random values that follow the standard uniform distribution $U(0,1)$. At each iteration, the endmember vector is standardized by dividing by its maximum for uniqueness. The

algorithm stops when the relative change of the standardized endmember $\widetilde{\mathbf{m}}$ between the $(t-1)$-th and the t-th iterations given by

$$\frac{\|\widetilde{\mathbf{m}}^{(t)} - \widetilde{\mathbf{m}}^{(t-1)}\|_2^2}{\|\widetilde{\mathbf{m}}^{(t-1)}\|_2^2}$$

is less than a small threshold value.

Input: A reference image $\mathbf{Y_m} \in \mathbb{R}_+^{C\times N}$;
Output: The standardized endmember vector $\widetilde{\mathbf{m}}$;
Initialization: $\mathbf{a}^{(0)}$, $\mathbf{m}^{(0)}$;
repeat

$a_n^{(t)} \leftarrow \widetilde{a}_n^{(t-1)} \cdot \left(\frac{\sum_c y_{cn}\widetilde{m}_c^{(t-1)}}{\widetilde{m}_c^{(t-1)}\widetilde{a}_n^{(t-1)}}\right) \Big/ \left(\sum_c \widetilde{m}_c^{(t-1)}\right)$;

$m_c^{(t)} \leftarrow \widetilde{m}_c^{(t-1)} \cdot \left(\frac{\sum_n y_{cn}a_n^{(t)}}{\widetilde{m}_c^{(t-1)}a_n^{(t)}}\right) \Big/ \left(\sum_n a_n^{(t)}\right)$;

$\widetilde{m}_c^{(t)} \leftarrow m_c^{(t)} / \|\mathbf{m}^{(t)}\|_\infty$;

$\widetilde{a}_n^{(t)} \leftarrow a_n^{(t)} \cdot \|\mathbf{m}^{(t)}\|_\infty$;

until *the stopping criterion is satisfied*;

Algorithm 1: Multiplicative update algorithm for PNMF (1)

We are now in a position to detail the proposed algorithm for solving the proposed regularized sparse and low-rank Poisson regression unmixing approach (SL-PRU) that reads

$$\min_{\mathbf{A}\in\mathbb{R}_+^{R\times N}} \mathbf{1}_C^\top \left[\mathbf{MA} - \mathbf{Y} \circ \log\left(\mathbf{MA}\right)\right] \mathbf{1}_N + \lambda_1 \|\mathbf{A}\|_{\mathbf{w}_p,*} + \lambda_2 \|\mathbf{W}_q\mathbf{A}\|_{2,1}. \tag{2}$$

Inspired by the work in Giampouras *et al.* (2016), an alternating direction method of multipliers (ADMM) technique (Boyd *et al.*, 2011) is adopted in our study by first letting all elements of $\mathbf{w}_p$ be equal to ensure the convexity of the low-rankness regularization term in SL-PRU (2). Similar to the work in Giampouras *et al.* (2016), we introduce auxiliary variables $\mathbf{V}_1 \in \mathbb{R}^{C\times N}$, $\mathbf{V}_2, \mathbf{V}_3, \mathbf{V}_4 \in \mathbb{R}^{R\times N}$ and reformulate SL-PRU (2) as follows

$$\begin{aligned} \min_{\mathbf{U},\mathbf{V}_1,\mathbf{V}_2,\mathbf{V}_3,\mathbf{V}_4} \quad & \mathbf{1}_C^\top \left[\mathbf{V}_1 - \mathbf{Y} \circ \log\left(\mathbf{V}_1\right)\right] \mathbf{1}_N + \lambda_1 \|\mathbf{V}_2\|_{\mathbf{w}_p,*} + \lambda_2 \|\mathbf{W}_q\mathbf{V}_3\|_{2,1} + \mathcal{I}_{\mathbb{R}_+}(\mathbf{V}_4), \\ \text{s.t.} \quad & \mathbf{V}_1 = \mathbf{MU}, \mathbf{V}_2 = \mathbf{U}, \mathbf{V}_3 = \mathbf{U}, \mathbf{V}_4 = \mathbf{U}, \end{aligned} \tag{3}$$

where $\mathcal{I}_{\mathbb{R}_+}(\cdot)$ is the indicator function which is zero if all the entries are nonnegative and infinity otherwise. The augmented Lagrangian function for the constrained optimization problem (3) is given as follows:

$$\begin{aligned} &\mathcal{L}_1(\mathbf{U}, \mathbf{V}_1, \mathbf{V}_2, \mathbf{V}_3, \mathbf{V}_4, \mathbf{D}_1, \mathbf{D}_2, \mathbf{D}_3, \mathbf{D}_4) \\ &= \mathbf{1}_C^\top \left[\mathbf{V}_1 - \mathbf{Y} \circ \log\left(\mathbf{V}_1\right)\right] \mathbf{1}_N + \lambda_1 \|\mathbf{V}_2\|_{\mathbf{w}_p,*} + \lambda_2 \|\mathbf{W}_q\mathbf{V}_3\|_{2,1} + \mathcal{I}_{\mathbb{R}_+}(\mathbf{V}_4) \\ &\quad + \operatorname{tr}(\mathbf{D}_1^\top(\mathbf{V}_1 - \mathbf{MU})) + \operatorname{tr}(\mathbf{D}_2^\top(\mathbf{V}_2 - \mathbf{U})) + \operatorname{tr}(\mathbf{D}_3^\top(\mathbf{V}_3 - \mathbf{U})) + \operatorname{tr}(\mathbf{D}_4^\top(\mathbf{V}_4 - \mathbf{U})) \\ &\quad + \frac{\mu}{2}\left(\|\mathbf{MU} - \mathbf{V}_1\|_F^2 + \|\mathbf{U} - \mathbf{V}_2\|_F^2 + \|\mathbf{U} - \mathbf{V}_3\|_F^2 + \|\mathbf{U} - \mathbf{V}_4\|_F^2\right), \end{aligned} \tag{4}$$

where $\mathbf{D}_1 \in \mathbb{R}^{C\times N}$, $\mathbf{D}_2, \mathbf{D}_3, \mathbf{D}_4 \in \mathbb{R}^{R\times N}$ denote the Lagrange multipliers, $\operatorname{tr}(\cdot)$ denotes matrix trace, $\mu > 0$ is a Lagrange multiplier regularization parameter, and $\|\cdot\|_F$ denotes the Frobenius norm.

Denoting the identity matrix of size $k \times k$ as $\mathbf{I}_k$ and the scaled Lagrange multipliers as $\mathbf{D}'_i = \mathbf{D}_i/\mu, i = 1, 2, 3, 4$, the augmented Lagrangian function $\mathcal{L}_1$ can be rewritten as

$$\begin{aligned}\mathcal{L}_2(\mathbf{U}, \mathbf{V}, \mathbf{D}) = &\mathbf{1}_C^\top \left[\mathbf{V}_1 - \mathbf{Y} \circ \log\left(\mathbf{V}_1\right)\right] \mathbf{1}_N + \lambda_1 \|\mathbf{V}_2\|_{\mathbf{w}_p,*} + \lambda_2 \|\mathbf{W}_q \mathbf{V}_3\|_{2,1} + \mathcal{I}_{\mathbb{R}_+}(\mathbf{V}_4) \\ &+ \frac{\mu}{2}\|\mathbf{G}\mathbf{U} + \mathbf{B}\mathbf{V} - \mathbf{D}\|_F^2,\end{aligned} \tag{5}$$

where

$$\mathbf{V} = \begin{bmatrix}\mathbf{V}_1\\ \mathbf{V}_2\\ \mathbf{V}_3\\ \mathbf{V}_4\end{bmatrix}, \mathbf{D} = \begin{bmatrix}\mathbf{D}'_1\\ \mathbf{D}'_2\\ \mathbf{D}'_3\\ \mathbf{D}'_4\end{bmatrix}, \mathbf{G} = \begin{bmatrix}\mathbf{M}\\ \mathbf{I}_R\\ \mathbf{I}_R\\ \mathbf{I}_R\end{bmatrix}, \mathbf{B} = -\mathbf{I}_{C+3R}.$$

The proposed ADMM-type algorithm for solving SL-PRU sequentially optimizes (4) or (5) with respect to each variable while the other variables remain as the latest values. Note that the augmented Lagrangian (5) is convex w.r.t. $\mathbf{U}$, $\mathbf{V}_1$, $\mathbf{V}_2$, $\mathbf{V}_3$, and $\mathbf{V}_4$, respectively, due to the assumption that all elements of $\mathbf{w}_p$ are equal and the fact that all entries of $\mathbf{W}_q$ are nonnegative. Therefore, at the t-th iteration, the updates of the abundance matrix $\mathbf{U}$ and the auxiliary variables $\mathbf{V}_1$, $\mathbf{V}_2$, $\mathbf{V}_3$, and $\mathbf{V}_4$ can be deduced, respectively, as follows:

Updating $\mathbf{U}$: The minimization of $\mathcal{L}_2$ w.r.t. $\mathbf{U}$ at the t-th iteration is equivalent to

$$\frac{\partial \frac{\mu}{2}\|\mathbf{G}\mathbf{U} + \mathbf{B}\mathbf{V}^{(t-1)} - \mathbf{D}\|_F^2}{\partial \mathbf{U}} = 0,$$

which yields

$$\mu \mathbf{G}^\top(\mathbf{G}\mathbf{U} + \mathbf{B}\mathbf{V} - \mathbf{D}) = \mu\left[\left(\mathbf{M}^\top\mathbf{M} + 3\mathbf{I}_R\right)\mathbf{U} - \mathbf{G}^\top(\mathbf{B}\mathbf{V} - \mathbf{D})\right] = 0.$$

As a result, we have

$$\begin{aligned}\mathbf{U}^{(t)} = &\arg\min_{\mathbf{U}} \mathcal{L}_2\left(\mathbf{U}, \mathbf{V}^{(t-1)}, \mathbf{D}^{(t-1)}\right) = \left(\mathbf{M}^\top\mathbf{M} + 3\mathbf{I}_R\right)^{-1} \\ &\left[\mathbf{M}^\top\left(\mathbf{V}_1^{(t-1)} + \mathbf{D}_1^{'(t-1)}\right) + \mathbf{V}_2^{(t-1)} + \mathbf{D}_2^{'(t-1)} + \mathbf{V}_3^{(t-1)} + \mathbf{D}_3^{'(t-1)} + \mathbf{V}_4^{(t-1)} + \mathbf{D}_4^{'(t-1)}\right].\end{aligned}$$

Updating $\mathbf{V}_1$: Denoting the (c, n)-th entry of $\mathbf{V}_1$ as v_{cn} with $c = 1, \cdots, C$ and $n = 1, \cdots, N$, we have

$$\mathbf{1}_C^\top \left[\mathbf{V}_1 - \mathbf{Y} \circ \log\left(\mathbf{V}_1\right)\right] \mathbf{1}_N = \sum_{c,n}[v_{cn} - y_{cn}\log(v_{cn})].$$

Since

$$\left(\frac{\partial \mathbf{1}_C^\top \left[\mathbf{V}_1 - \mathbf{Y} \circ \log\left(\mathbf{V}_1\right)\right] \mathbf{1}_N}{\partial \mathbf{V}_1}\right)_{cn} = \frac{\partial[v_{cn} - y_{cn}\log(v_{cn})]}{\partial v_{cn}} = 1 - \frac{y_{cn}}{v_{cn}},$$

and

$$\left(\frac{\partial \frac{\mu}{2}\|\mathbf{M}\mathbf{U}^{(t)} - \mathbf{V}_1 - \mathbf{D}'_1\|_F^2}{\partial \mathbf{V}_1}\right)_{cn} = \mu[v_{cn} + (\mathbf{D}_1^{'(t-1)} - \mathbf{M}\mathbf{U}^{(t)})_{cn}],$$

we know that minimizing $\mathcal{L}_2$ w.r.t. v_{cn} is equivalent to

$$v_{cn}^2 - (\mathbf{V}_1'^{(t)})_{cn} \cdot v_{cn} - \frac{y_{cn}}{\mu} = 0,$$

where $\mathbf{V}_1'^{(t)} = \mathbf{M}\mathbf{U}^{(t)} - \mathbf{D}_1^{'(t-1)} - 1/\mu$. Solving the above quadratic equation w.r.t. v_{cn}, we have

$$\mathbf{V}_1^{(t)} = \arg\min_{\mathbf{V}_1} \mathcal{L}_2 \left(\mathbf{U}^{(t)}, \begin{bmatrix} \mathbf{V}_1 \\ \mathbf{V}_2^{(t-1)} \\ \mathbf{V}_3^{(t-1)} \\ \mathbf{V}_4^{(t-1)} \end{bmatrix}, \mathbf{D}^{(t-1)} \right) = \frac{\mathbf{V}_1'^{(t)} + \sqrt{\mathbf{V}_1'^{(t)} \circ \mathbf{V}_1'^{(t)} + 4\mathbf{Y}/\mu}}{2},$$

where $\sqrt{\cdot}$ denotes the element-wise square root.

Updating $\mathbf{V}_2$: Minimizing $\mathcal{L}_2$ w.r.t. $\mathbf{V}_2$ is equivalent to

$$\min_{\mathbf{V}_2} \lambda_1 \|\mathbf{V}_2\|_{\mathbf{w}_p,*} + \frac{\mu}{2}\|\mathbf{U} - \mathbf{V}_2 - \mathbf{D}_2'\|_F^2,$$

which can be solved by a soft-thresholding operation (Cai *et al.*, 2010) on the singular values of $\mathbf{V}_2$. Recall that the singular value decomposition of $\mathbf{U}^{(t)} - \mathbf{D}_2^{'(t-1)}$ is $\mathbf{S}_l \mathbf{\Sigma}^{(t)} \mathbf{S}_r^\top$, the soft-thresholding function on each diagonal element of $\mathbf{\Sigma}^{(t)}$, i.e., $\sigma_p^{(t)}$, with parameter $\lambda_1 \mathbf{w}_p/\mu$ is

$$\max\{\mathbf{0}, \sigma_p^{(t)} - \lambda_1 \mathbf{w}_p/\mu\},$$

for any $p = 1, 2, \cdots, \mathrm{rank}(\mathbf{V}_2)$. Thus, the optimization w.r.t. $\mathbf{V}_2$ gives

$$\mathbf{V}_2^{(t)} = \arg\min_{\mathbf{V}_2} \mathcal{L}_2 \left(\mathbf{U}^{(t)}, \begin{bmatrix} \mathbf{V}_1^{(t)} \\ \mathbf{V}_2 \\ \mathbf{V}_3^{(t-1)} \\ \mathbf{V}_4^{(t-1)} \end{bmatrix}, \mathbf{D}^{(t-1)} \right) = \mathbf{S}_l[\mathrm{sign}(\mathbf{\Sigma}^{(t)}) \circ \max\{\mathbf{0}, \mathbf{\Sigma}^{(t)} - \lambda_1 \mathrm{diag}(\mathbf{w}_p)/\mu\}]\mathbf{S}_r^\top,$$

where $\mathrm{sign}(\cdot)$ is the element-wise sign function, $\max\{\cdot,\cdot\}$ denotes the element-wise max function, and $\mathrm{diag}(\cdot)$ creates a matrix with diagonal elements equal to the vector elements.

Updating $\mathbf{V}_3$: Minimizing $\mathcal{L}_2$ w.r.t. $\mathbf{V}_3$ is equivalent to

$$\min_{\mathbf{V}_3} \lambda_2 \|\mathbf{W}_q \mathbf{V}_3\|_{2,1} + \frac{\mu}{2}\|\mathbf{U} - \mathbf{V}_3 - \mathbf{D}_3'\|_F^2,$$

which can be solved by a vectorial soft-thresholding operation (Wright *et al.*, 2009) on each row of $\mathbf{V}_3^{(t)}$. More specifically, denoting $\mathbf{V}_{3,r}^{(t)}$ as the r-th row of $\mathbf{V}_3^{(t)}$ and $\mathbf{x}_r^{(t)}$ as the r-th row of $\mathbf{U}^{(t)} - \mathbf{D}_3^{'(t-1)}$ where $r = 1, \ldots, R$, each row of $\mathbf{V}_3$ is updated sequentially as

$$\mathbf{V}_{3,r}^{(t)} = \arg\min_{\mathbf{V}_{3,r}} \mathcal{L}_2 \left(\mathbf{U}^{(t)}, \begin{bmatrix} \mathbf{V}_1^{(t)} \\ \mathbf{V}_2^{(t)} \\ \mathbf{V}_{3,1}^{(t)} \\ \vdots \\ \mathbf{V}_{3,r-1}^{(t)} \\ \mathbf{V}_{3,r} \\ \mathbf{V}_{3,r+1}^{(t-1)} \\ \vdots \\ \mathbf{V}_{3,R}^{(t-1)} \\ \mathbf{V}_4^{(t-1)} \end{bmatrix}, \mathbf{D}^{(t-1)} \right) = \frac{\mathbf{x}_r^{(t)} \max\{\|\mathbf{x}_r^{(t)}\|_2 - \lambda_2 w_{q,r}/\mu, 0\}}{\max\{\|\mathbf{x}_r^{(t)}\|_2 - \lambda_2 w_{q,r}/\mu, 0\} + \lambda_2 w_{q,r}/\mu}.$$

Input: The data matrix $\mathbf{Y} \in \mathbb{R}_+^{C\times N}$, and the endmember matrix $\mathbf{M} \in \mathbb{R}_+^{C\times R}$;
Output: The abundance matrix $\mathbf{U}$;
Initialization: $\mathbf{U}^0, \mathbf{V}_i^0, \mathbf{D}_i^0, \quad i = 1, 2, 3, 4$;
repeat

$\mathbf{U}^{(t)} \leftarrow \left(\mathbf{M}^\top\mathbf{M} + 3\mathbf{I}_R\right)^{-1}\Big[\mathbf{M}^\top\left(\mathbf{V}_1^{(t-1)} + \mathbf{D}_1^{'(t-1)}\right) + \mathbf{V}_2^{(t-1)} + \mathbf{D}_2^{'(t-1)} + \mathbf{V}_3^{(t-1)} + \mathbf{D}_3^{'(t-1)} + \mathbf{V}_4^{(t-1)} + \mathbf{D}_4^{'(t-1)}\Big]$;

$\mathbf{V}_1^{(t)} \leftarrow \left(\mathbf{V}_1^{(t)} + \sqrt{\mathbf{V}_1^{(t)} \circ \mathbf{V}_1^{(t)} + 4\mathbf{Y}/\mu}\right)\Big/2$;

$\mathbf{V}_2^{(t)} \leftarrow \mathbf{S}_l[\text{sign}(\mathbf{\Sigma}) \circ \max\{\mathbf{0}, \mathbf{\Sigma} - \lambda_1\text{diag}(\mathbf{w}_p)/\mu\}]\mathbf{S}_r^\top$;

$\mathbf{V}_{3,r}^{(t)} \leftarrow (\mathbf{x}_r^{(t)}\max\{\|\mathbf{x}_r^{(t)}\|_2 - \lambda_2 w_{q,r}/\mu, 0\})/(\max\{\|\mathbf{x}_r^{(t)}\|_2 - \lambda_2 w_{q,r}/\mu, 0\} + \lambda_2 w_{q,r}/\mu)$, $r = 1, \ldots, R$;

$\mathbf{V}_4^{(t)} \leftarrow \max\left\{\mathbf{U}^{(t)} - \mathbf{D}_4^{\prime(t-1)}, \mathbf{0}\right\}$;

$\mathbf{D}_1^{\prime(t)} \leftarrow \mathbf{D}_1^{\prime(t-1)} - \mathbf{M}\mathbf{U}^{(t)} + \mathbf{V}_1^{(t)}$;

$\mathbf{D}_i^{\prime(t)} \leftarrow \mathbf{D}_i^{\prime(t-1)} - \mathbf{U}^{(t)} + \mathbf{V}_i^{(t)}, \quad i = 2, 3, 4$;

until *the stopping criteria are satisfied*;

Algorithm 2: The proposed ADMM-type algorithm for SL-PRU (2)

Updating $\mathbf{V}_4$: Minimizing $\mathcal{L}_2$ w.r.t. $\mathbf{V}_4$ is equivalent to

$$\min_{\mathbf{V}_4} \mathcal{I}_{\mathbb{R}_+}(\mathbf{V}_4) + \frac{\mu}{2}\|\mathbf{U} - \mathbf{V}_4 - \mathbf{D}_4'\|_F^2,$$

where the first term is to project $\mathbf{V}_4$ onto the nonnegative orthant. Thus, the optimization w.r.t. $\mathbf{V}_4$ gives

$$\mathbf{V}_4^{(t)} = \arg\min_{\mathbf{V}_4} \mathcal{L}_2\left(\mathbf{U}^{(t)}, \begin{bmatrix}\mathbf{V}_1^{(t)}\\ \mathbf{V}_2^{(t)}\\ \mathbf{V}_3^{(t)}\\ \mathbf{V}_4\end{bmatrix}, \mathbf{D}^{(t-1)}\right) = \max\left\{\mathbf{U}^{(t)} - \mathbf{D}_4^{'(t-1)}, \mathbf{0}\right\}.$$

Updating $\mathbf{D}_1'$, $\mathbf{D}_2'$, $\mathbf{D}_3'$, and $\mathbf{D}_4'$: These scaled Lagrange multipliers are updated as follows

$$\mathbf{D}_1^{\prime(t)} = \mathbf{D}_1^{\prime(t-1)} - \mathbf{M}\mathbf{U}^{(t)} + \mathbf{V}_1^{(t)}, \quad \mathbf{D}_i^{\prime(t)} = \mathbf{D}_i^{\prime(t-1)} - \mathbf{U}^{(t)} + \mathbf{V}_i^{(t)}, \quad i = 2, 3, 4.$$

The stopping criteria adopted in the algorithm are based on the primal and dual residuals (Boyd *et al.*, 2011) $\mathbf{r}_p$ and $\mathbf{r}_d$ given by

$$\mathbf{r}_p = \mathbf{G}\mathbf{U}^{(t)} + \mathbf{B}\mathbf{V}^{(t)}, \quad \mathbf{r}_d = \mu\mathbf{G}^\top\mathbf{B}\left(\mathbf{V}^{(t)} - \mathbf{V}^{(t-1)}\right),$$

that go to 0, respectively, as $t \to \infty$. The algorithm terminates whenever any of the ℓ_2 norms of $\mathbf{r}_p$ or $\mathbf{r}_d$ is less than a small threshold value or some number of iterations is reached. To enhance the performance of the algorithm, as done in Giampouras *et al.* (2016), we also update the weights $\mathbf{w}_p$ and $\mathbf{W}_q$ based on $\mathbf{U}^{(t)}$ at the t-th iteration as follows

$$w_{p,i}^{(t)} = \frac{1}{\sigma_i(\mathbf{U}^{(t)}) + \varepsilon}, \quad w_{q,r} = \frac{1}{\|\mathbf{u}_r^{(t)}\|_2 + \varepsilon},$$

where $\mathbf{u}_r^{(t)}$ denotes the r-th row of $\mathbf{U}^{(t)}$ and $\varepsilon > 0$ is assigned a small value to avoid singularities.

The pseudo-code of the proposed algorithm for solving SL-PRU is presented in Algorithm 2.

2 Supplementary Table 1: Salient Characteristics of FISH Probes

Probe name	Target	Sequence	Fluorophore in plaque smear image	Reference
ACT-476	Actinomyces	ATCCAGCTACCGTCAACC	Alexafluor 488	(Gmür and Lüthi-Schaller, 2007)
STR-405	Streptococcus	TAGCCGTCCCTTTCTGGT	Alexafluor 594	(Paster, Bruce J *et al.*, 1998)
FUS-714	Fusobacterium	GGCTTCCCCATCGGCATT	Alexafluor 555	(Valm, Alex M *et al.*, 2011)
LEP-568	Leptotrichia	GCCTAGATGCCCTTTATG	Alexafluor 660	(Valm, Alex M *et al.*, 2011)
NEI-1030	Neisseriaceae	CCTGTGTTACGGCTCCCG	Tetrachlorofluorescein	(Valm, Alex M *et al.*, 2011)
PGI-350	Porphyromonas	CCTCACGCCTTACGACGG	Alexafluor 647	(Valm, Alex M *et al.*, 2011)
VEI-488	Veillonella	CCGTGGCTTTCTATTCCG	Alexafluor 514	(Chalmers *et al.*, 2008)
PRV-392	Prevotella	GCACGCTACTTGGCTGG	Alexafluor 633	(Diaz *et al.*, 2006)
PAS-111	Pasteurellaceae	TCCCAAGCATTACTCACC	Rhodamine Red-X	(Valm, Alex M *et al.*, 2011)
EUB-338	All bacteria	GCTGCCTCCCGTAGGAGT	Used for E. coli reference standards	(Amann *et al.*, 1990)

Supplementary Table 1: Salient characteristics of all FISH probes used in this study

3 Supplementary Figure 1: Emission Spectra of Endmembers and the Extracted Endmembers

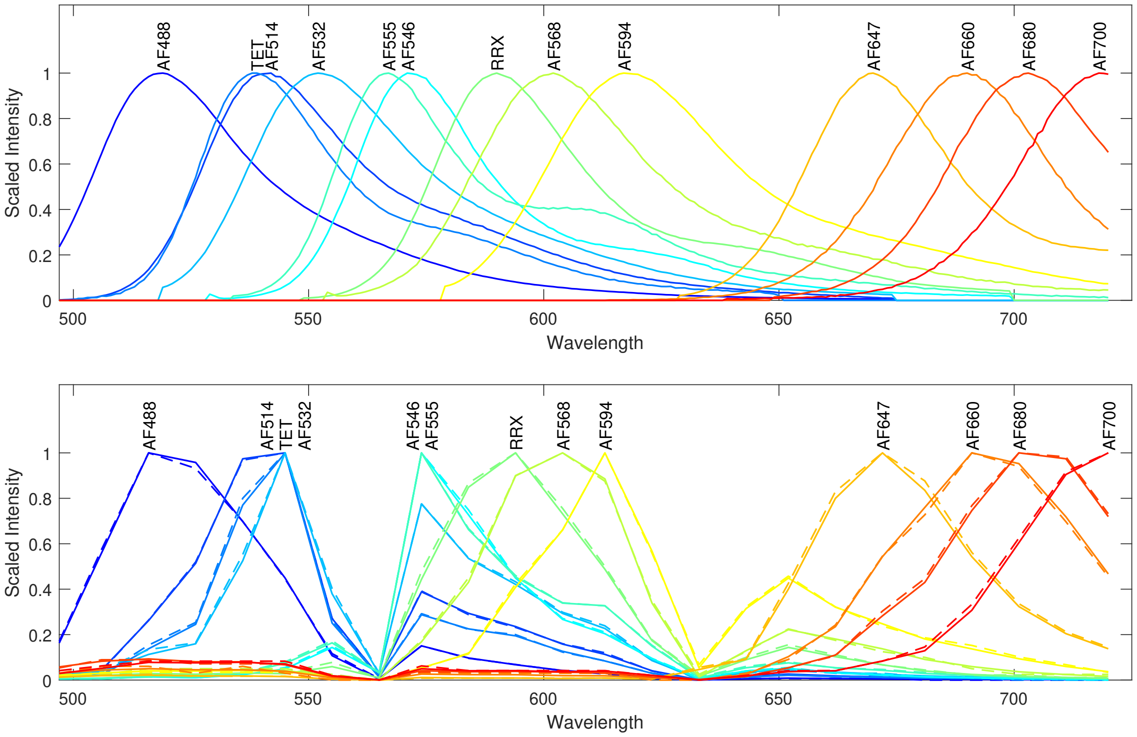

Supplementary Figure 1: Empirically measured spectra of thirteen endmembers from Supplementary Table 2 above. Top row: Fluorometer data provided by the manufacturers of the dyes sampled in 1 nm wavelength bands. Bottom row: Endmembers extracted by arithmetic mean (dashed curves) method and PNMF (solid curves) from real images of labeled E. coli acquired on a spectral confocal microscope with 9.8 nm wavelength bands.

4 Supplementary Table 2: Descriptions of Fluorophores

Fluorophore	Abbreviation	Peak Excitation λ (nm)	Peak Emission λ (nm)
Alexafluor 488	AF488	495	519
Alexafluor 514	AF514	517	542
Tetrachlorofluorescein	TET	522	539
Alexafluor 532	AF532	532	553
Alexafluor 546	AF546	556	573
Alexafluor 555	AF555	555	565
Rhodamine Red-X	RRX	560	580
Alexafluor 568	AF568	578	603
Alexafluor 633	AF633	621	639
Alexafluor 647	AF647	650	665
Alexafluor 660	AF660	663	690
Alexafluor 680	AF680	679	702
Alexafluor 700	AF700	702	723

Supplementary Table 2: Names, abbreviations, and peak excitation and emission wavelengths for all fluorophores used in this study.

5 Supplementary Figure 2: Quantitative Line Scan Analysis on Dental Plaque Smear Images

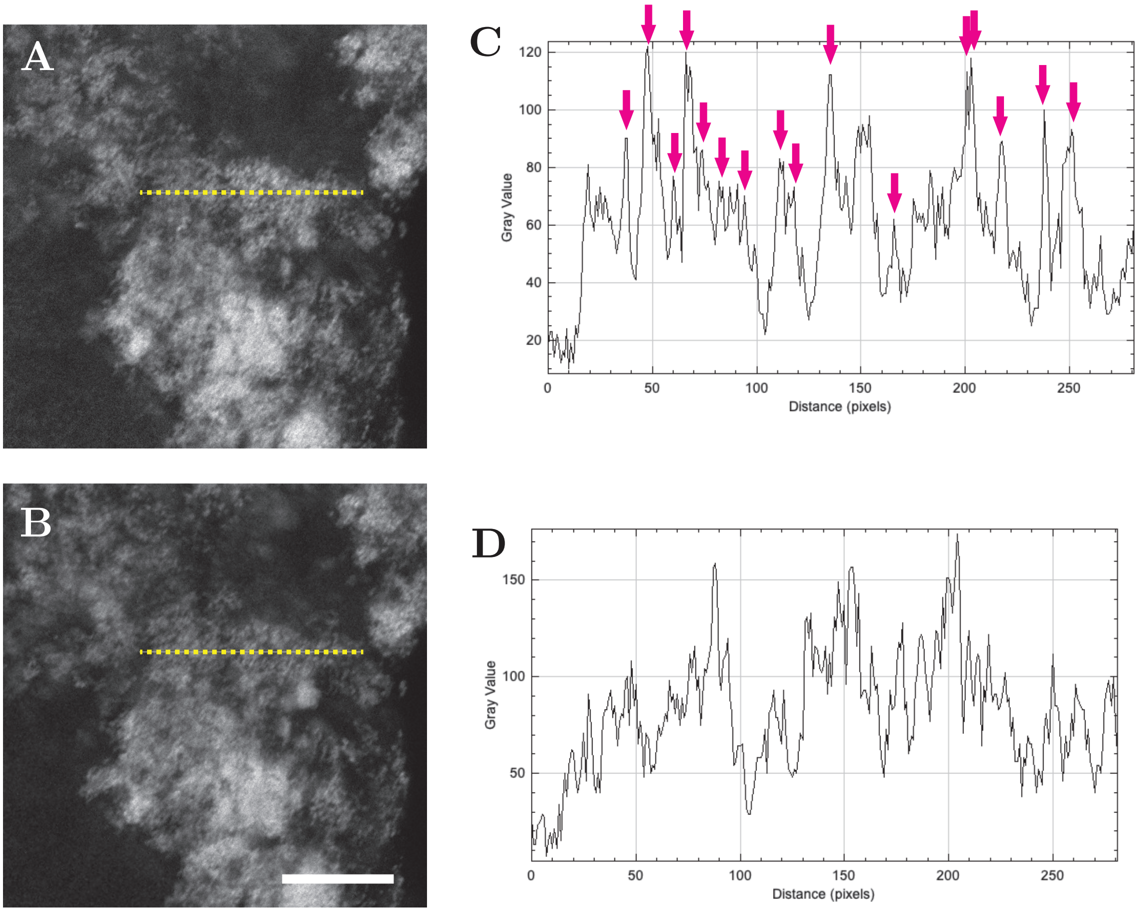

Supplementary Figure 2: A-B. Region of interest images from the Streptococcus (Alexa-fluor 594) channel from (A) SL-PRU unmixed image and (B) commercial least squares unmixed image. Yellow dotted lines show where line scan analysis was performed in each image. Bar = 25 µm. C-D: Line scan analysis results (intensity vs. pixel position) for SL-PRU unmixing (C) and least squares unmixing (D) along the length of the lines in A & B. Magenta arrows in (C) identify peaks with a full-width-at-half-maximum of approximately 4-5 pixels (0.7-0.9 µm), the known diameter of oral Streptococcus cells (Baron, Samuel and Patterson, Maria Jevitz, 1996).

6 Supplementary Movie: SL-RPU Unmixed Dental Plaque Smear

3-D volume-rendered movie of a dental plaque smear labeled with 8 taxon-specific FISH probes (See Figure 6 in Main Text for color legend). Spectral image was unmixed with SL-RPU.

References

Amann, R. I. *et al.* (1990). Combination of 16s rRNA-targeted oligonucleotide probes with flow cytometry for analyzing mixed microbial populations. *Applied and Environmental Microbiology*, **56**(6), 1919–1925.

Baron, Samuel and Patterson, Maria Jevitz (1996). Streptococcus. *Clinical Microbiology Reviews*, **2**(3), 315.

Boyd, S. *et al.* (2011). Distributed optimization and statistical learning via the alternating direction method of multipliers. *Foundations and Trends® in Machine Learning*, **3**(1), 1–122.

Cai, J.-F. *et al.* (2010). A singular value thresholding algorithm for matrix completion. *SIAM Journal on Optimization*, **20**(4), 1956–1982.

Chalmers, N. I. *et al.* (2008). Characterization of a Streptococcus sp.-Veillonella sp. community micromanipulated from dental plaque. *Journal of Bacteriology*, **190**(24), 8145–8154.

Diaz, P. I. *et al.* (2006). Molecular characterization of subject-specific oral microflora during initial colonization of enamel. *Applied and Environmental Microbiology*, **72**(4), 2837–2848.

Giampouras, P. V. *et al.* (2016). Simultaneously sparse and low-rank abundance matrix estimation for hyperspectral image unmixing. *IEEE Transactions on Geoscience and Remote Sensing*, **54**(8), 4775–4789.

Gmür, R. and Lüthi-Schaller, H. (2007). A combined immunofluorescence and fluorescent in situ hybridization assay for single cell analyses of dental plaque microorganisms. *Journal of Microbiological Methods*, **69**(2), 402–405.

Lee, D. and Seung, H. S. (2000). Algorithms for non-negative matrix factorization. *Advances in Neural Information processing Systems*, **13**.

Paster, Bruce J *et al.* (1998). Identification of oral streptococci using PCR-based, reverse-capture, checkerboard hybridization. *Methods in Cell Science*, **20**(1), 223–231.

Valm, Alex M *et al.* (2011). Systems-level analysis of microbial community organization through combinatorial labeling and spectral imaging. *Proceedings of the National Academy of Sciences of the United States of America*, **108**(10), 4152–4157.

Wright, S. J. *et al.* (2009). Sparse reconstruction by separable approximation. *IEEE Transactions on Signal Processing*, **57**(7), 2479–2493.
